# The *Radish* Gene Reveals a Memory Component with Variable Temporal Properties

**DOI:** 10.1371/journal.pone.0024557

**Published:** 2011-09-02

**Authors:** Holly LaFerriere, Katherine Speichinger, Astrid Stromhaug, Troy Zars

**Affiliations:** Division of Biological Sciences, University of Missouri, Columbia, Missouri, United States of America; Harvard University, United States of America

## Abstract

Memory phases, dependent on different neural and molecular mechanisms, strongly influence memory performance. Our understanding, however, of how memory phases interact is far from complete. In *Drosophila*, aversive olfactory learning is thought to progress from short-term through long-term memory phases. Another memory phase termed anesthesia resistant memory, dependent on the *radish* gene, influences memory hours after aversive olfactory learning. How does the *radish*-dependent phase influence memory performance in different tasks? It is found that the *radish* memory component does not scale with the stability of several memory traces, indicating a specific recruitment of this component to influence different memories, even within minutes of learning.

## Introduction

Memory phases strongly influence memory performance over time. These phases can be influenced by different neural structures and molecular mechanisms. In the honeybee, for example, the role of the cAMP/PKA cascade is required in a short time window after training to induce a long-term memory phase [1]. And in vertebrate animals, it is widely held that the vertebrate hippocampus is needed in a transient way for some new memories to be formed [2]. Our understanding, however, of how memory phases ultimately influence conditioned behavior is far from complete.

Current models of how memory phases influence memory performance in *Drosophila* largely depends on results from aversive olfactory learning. In this type of learning flies are conditioned by associating electric shock with an odorant. A memory test allows flies to choose between the shock-associated odorant and a second odorant not previously associated with shock [3]. Memory performance in this paradigm is thought to mature through short-term memory (STM), middle-term memory (MTM), and long-term memory (LTM) phases [4], [5]. Memory in the minutes range after training is influenced by the cAMP / PKA signaling cascade, among other genes [3], [6]–[9]. An intriguing memory phase termed anesthesia resistant memory (ARM) has also been identified, which develops within hours of learning, and is operationally defined as the memory component that is resistant to the effects of cold-shock induced anesthesia [4]. The *radish* (*rsh*) gene (formerly CG15720, now referred to as CG42628) provides the main molecular insight into the mechanisms of ARM [4], [10]–[13]. While mutation of the *rsh* gene leads to a minor reduction in memory performance shortly after aversive olfactory training [13], this gene plays an increasingly important role in memory performance as ARM provides a more significant component of the overall memory [4], [13]. Thus, the currently accepted model is that memory consolidation in the range of hours after training is critically regulated by a *rsh*-dependent ARM.

We asked whether the *rsh* gene influence on memory formation is restricted to an hours-long memory phase following other forms of learning. Appetitive olfactory learning and operant place conditioning induce memories that decay at different rates compared to aversive classical olfactory learning. Aversive olfactory memory after one training session decays to near-zero levels within 24 hrs [3], [14]. In contrast, appetitive olfactory learning that associates sugar with an odorant in a single training session leads to memory that is stable for at least 24 hrs [15]–[17]. In operant place learning, where individual flies are conditioned to avoid part of a long narrow chamber using high temperature as a negative reinforcer, short training leads to a memory that decays within minutes [18], [19]. With prolonged intermittent-training memory decays to negligible levels within 2 hrs [20]. We asked how the *rsh*-dependent memory is established under these learning conditions.

## Results

**Figure 1:**
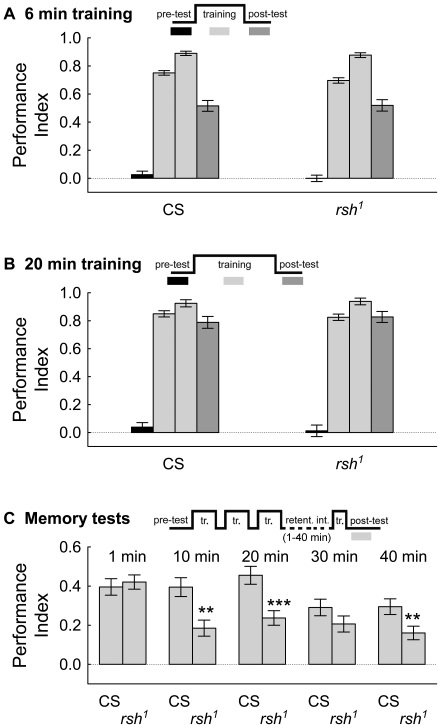
Mutation of the *rsh* gene does not influence conditioning or place memory tested directly after training. Following a 30 s pre-test period (black bars), wild-type CS and *rsh^1^* mutant flies were trained in two equal length periods for a total of either 6 or 20 min with 41°C (light gray bars). A 3 min memory was tested directly following in the post-test period (dark gray bars). The training, retention intervals, and testing patterns (both pre and post) are diagrammed for each panel, the time axis is not to scale. (*A*), Conditioning and memory tests were similar between the genotypes with 6 min of training (N = 331; pre-test: U = 12753.5, z = 1.07, P = 0.28; 1^st^ training period: U = 11877.0, z = 2.08, P = 0.04; 2^nd^ training period: U = 12888.5, z = 0.92, P = 0.36; post-test: U = 13237.0, z = 0.51, P = 0.61). (*B*) Conditioning and memory tests were also similar between the genotypes with 20 min of training (N = 232; pre-test: U = 6106.5, z = 1.22, P = 0.22; 1^st^ training period: U = 5740.5, z = 1.93, P = 0.06; 2^nd^ training period: U = 5802.0, z = −1.81,  = 0.07; post-test: U = 6463.0, z = −0.52, P = 0.60). (*C*) The *rsh* gene is necessary for normal short-term place memory. Flies were trained with intermittent training and then held for varying times (1 – 40 min) before being tested for memory with a short reminder training. The *rsh^1^* flies had memory performance similar to wild-type CS levels with a 1 min delay between training and the memory test (N = 447, U = 24641.5, z = 0.24, P = 0.8). Significant differences were found at several time points following training (10 min: N = 295, U = 8637.0, z = .02, **  =  P<0.01; 20 min: N = 330, U = 10074.5, z = 3.95, ***  =  P<0.001; 30 min: N = 311, U = 10926.0, z = 1.45, P = 0.1; 40 min: N = 351, U = 12941.5, z = 2.48, **  =  P<0.01). The values are means and error bars represent s.e.m.

The role of the *rsh* gene in place learning and memory in the heat-box was examined. Both wild-type CS and *rsh^1^* mutant flies were trained for either 6 or 20 min and place memory was tested directly afterward. The CS flies were used for comparison as this strain best represents the genetic background of the *rsh^1^*flies [12]. The *rsh^1^* allele is either a strong hypomorphic or null allele as there is a stop codon toward the end of the coding region which reduces Rsh protein levels to below detection limits [12]. Only subtle differences were identified between flies of these genotypes in their avoidance behavior during training and in the memory post-test (Fig. 1A and B). Thus, consistent with previous tests of *rsh^1^* flies in aversive olfactory memory and visual pattern learning [13], [21], up to this point the *rsh* gene has a minor role in early stages of memory formation.

Because the memory trace rapidly decays in place learning, we thought that the *rsh* gene might be important in place memory consolidation shortly after training. Thus, wild-type CS and *rsh^1^* flies were conditioned with intermittent training, removed from the chambers for 1 to 40 min in the retention interval, and tested for memory after a short 1 min reminder training. As in the memory test directly after training, a test of memory after a 1 min delay did not reveal a difference between flies of these genotypes (Fig. 1C). Memory with a short retention interval and reminder training, however, does have the expected lower memory performance levels in wild-type CS flies compared to memory tested directly after training (Fig. 1B and C) [20], [22]. From 10 to 40 min after conditioning the memory levels are lower in *rsh^1^* flies compared to CS, although not significantly so at 30 min post-training. This latter case might indicate an interesting dynamic in memory processes in this time range. Nevertheless, these results show that the *rsh* gene is necessary to partially consolidate place memory several minutes after training.

**Figure 2:**
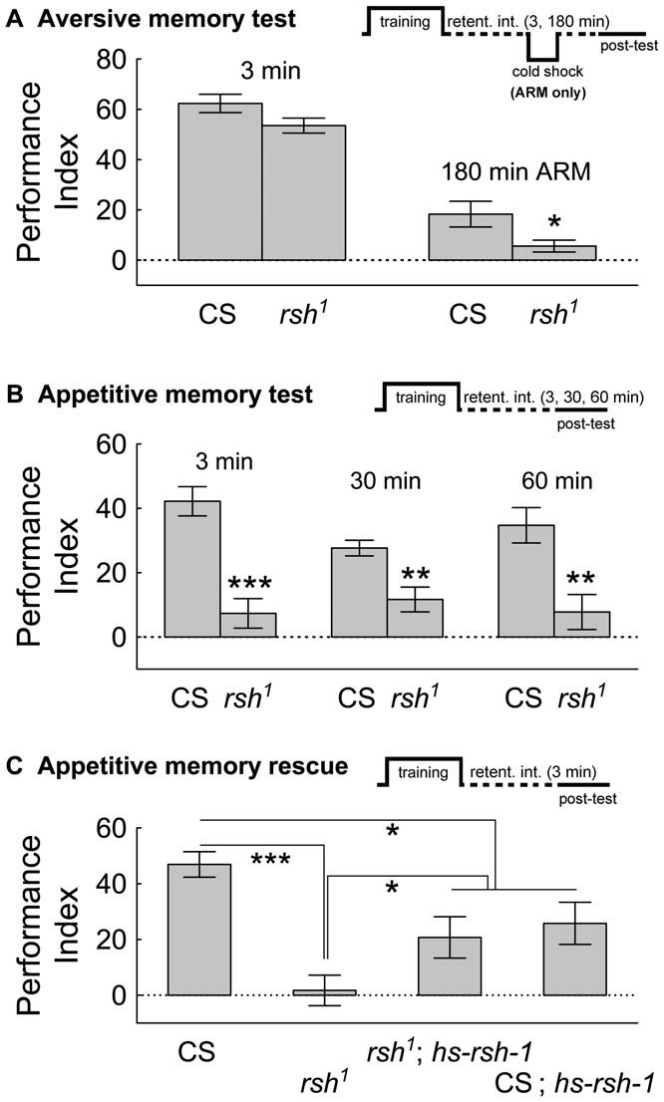
Mutation of the *rsh* gene reveals a major role in aversive olfactory memory (ARM) and is necessary for appetitive olfactory memory shortly after conditioning. Flies were either trained with odorants paired with electric shock or sugar reward. The training, cold-shock, retention intervals, and testing patterns (both pre and post) are diagrammed for each panel, the time axis is not to scale. (*A*) Olfactory memory tested three min after training is reduced in *rsh^1^* flies compared to CS flies, although levels do not reach statistical significance (F(1,12)  = 3.5, P = 0.09). To reveal the *rsh* function in aversive olfactory memory, wild-type CS and *rsh^1^* flies were trained with odorant / shock pairings, then after 2 hrs were given a cold-shock, memory was tested 1 hr later. Memory performance of *rsh^1^* flies was significantly lower than wild-type CS flies with this procedure (F(1,10)  = 5.0, *  =  P = 0.04). (*B*) Appetitive olfactory short-term memory was tested at 3, 30, and 60 min after the odorant / sucrose training session. A *rsh^1^* phenotype was evident at all tested time points after training (3 min: F(1,16)  = 29.2, ***  =  P<0.001; 30 min: F(1,14)  = 12.3, **  =  P<0.01; 60 min: F(1,14)  = 12.1, **  =  P<0.01). (*C*) The *rsh^1^* appetitive short term olfactory memory phenotype is rescued with a transgenic copy of the wild-type version of the *rsh* gene (F(3,32)  = 13.0, P<0.0001; post-hoc tests: CS vs *rsh^1^* ***  = P<0.001, *rsh^1^* vs. *rsh^1^*; hs-*rsh-1* *  = P<0.05, CS vs. *rsh^1^*; hs-*rsh-1*, *  = P<0.05; *rsh^1^* vs. CS; hs-*rsh-1* *  = P<0.05; CS vs. CS; hs-*rsh-1* *  = P<0.05). The values are means and error bars represent s.e.m.

We next examined the role of *rsh^1^* mutation in aversive olfactory memory as a way to affirm that we are indeed measuring *rsh*-dependent processes; a minor role in 3 min memory but a strong defect in ARM is a defining feature of the *rsh^1^* mutation [4], [13]. As found previously [12], *rsh^1^* mutant flies have a minor deficit in 3 min memory, but a significant ARM defect measured 3 hrs post-training (Fig. 2A).

That the *rsh* gene provides a memory consolidation function in a short time window for a memory which rapidly decays in the heat-box prompted examination of the *rsh* gene in appetitive olfactory memory performance. After training a memory was tested at several retention intervals. Surprisingly, we found that *rsh^1^* mutant flies had a severe deficit in appetitive memory as soon as we could measure the flies post-training (3 min) (Fig. 2B), and this deficit was still evident 60 min afterwards (a 180 min deficit of *rsh^1^* mutant flies was previously described) [16]. Partial rescue of the *rsh^1^* phenotype with expression of a wild-type version of the *rsh* gene (hs-*rsh-1*) indicates that the phenotypes we measured are a consequence of mutation of the *rsh* gene (Fig. 2C). In this case, no heat-shock was necessary to increase the memory performance levels of *rsh^1^* mutant flies to levels higher than *rsh^1^* mutant flies, and toward wild-type CS levels. Furthermore, addition of the hs-*rsh1* transgene does not improve wild-type CS flies memory levels which might have been the case if more *rsh* expression simply gave rise to higher memory performance. Indeed, slightly lower memory levels were found in CS, hs-*rsh1* flies compared to CS levels, suggesting there are optima in expression levels / domains for *rsh* function and olfactory memory formation. It is also possible that the partial rescue indicates that the phenotypes measured are partially dependent on mutation of the *rsh* gene. Nevertheless, that addition of the hs-*rsh1* transgene increases *rsh^1^* mutant flies appetitive olfactory memory levels provides strong evidence that *rsh*-dependent functions were measured.

Finally, we examined control behaviors of *rsh^1^* mutant and wild-type CS flies. The ability to avoid the odors used in conditioning of *rsh^1^* and rescued flies after starvation were similar to wild-type CS and other genetic control flies (Table 1). The olfactory tests used the same odorant concentrations and time allowed in the T-maze choice point as for conditioning. Furthermore, the ‘attractiveness’ of the sucrose used in the conditioning experiments was tested. The attractiveness of sucrose was tested in vials with a dried stripe of sucrose (the same concentration used in the conditioning experiment) [23]. The proportion of flies on the stripe over time was measured, and the average over two minutes was used as a sucrose responsivity measure. Differences in sucrose responsiveness in CS, *rsh^1^*, and other genetic control flies were not detected (Table 1), in contrast to previous findings in which a dilution of the sucrose reward was used to test for sucrose responsivity [17]. As a control for place learning we measured activity levels. The measure of activity is the average probability of moving in the pre-test period [24]. The activity levels were similar between wild-type CS and *rsh^1^* mutant flies (Table 1). Most importantly, normal conditioning and early place memory in *rsh^1^* flies suggests that they can sense and avoid the high temperatures used in the conditioning experiments (Fig. 1A). Thus, *rsh*–dependent changes in memory performance levels are independent of changes in olfactory, sugar-related, and temperature sensory defects, and locomotor activity differences.

**Table 1:** Control behaviors of wild-type CS and *rsh^1^* mutant flies.

Genotype	MCH avoidance (PI) N = 36	Oct avoidance (PI) N = 24	Sugar attractiveness N = 48	Activity (rel. units) N = 563
CS	20.6±5.2	12.7±5.2	0.61±0.04	0.73±0.01
*rsh^1^*	19.2±8.9	22.8±10.8	0.53±0.05	0.70±0.01
*rsh^1^*; hs*-rsh-1*	8.2±5.9	31.5±5.5	0.52±0.05	ND
*CS; hs-rsh-1*	25.8±7.9	33.1±9.7	0.55±0.04	ND

MCH avoidance: ANOVA F(3,32)  = 1.07, P = 0.4; Oct avoidance: F(3,20)  = 1.3, P = 0.3; Sugar attractiveness: ANOVA F(3,44) = 0.75, P = 0.53; Activity: F(1,561) = 3.3, P = 0.07.

## Discussion

Our results challenge the traditional view that the *rsh* gene provides a consolidation function for ARM in the range of hours after learning [4], [5]. The results described here reveal that the timing of *rsh* function depends on the learning context. In aversive place learning, with a memory trace that decays within hours, the *rsh* function is already evident within 10 min of conditioning. This is the first mutation that reduces place memory levels without also altering conditioned behavior during training. Furthermore, that *rsh^1^* flies have a phenotype in place memory indicates that *rsh* has a more general function in memory formation, which is not always the case [7], [25]–[28]. In appetitive olfactory learning, the *rsh* function is also critical for a memory tested within minutes of training. Remarkably, the appetitive memory is much more stable than either the place memory or the aversive olfactory memory [15], [16], [20]. Thus, one cannot simply scale the role of *rsh* with the stability of a memory trace, but the timing of the role of *rsh* in memory formation depends on the learning task.

The balance of memory phases or components that supports memory performance depends on conditioning parameters. One gains access to two primary memory components after aversive olfactory learning by training flies with either massed or spaced protocols. That is, with massed training of multiple training sessions, flies form a memory that is predominantly resistant to the effects of anesthesia (ARM) and sensitive to mutation of the *rsh* gene [4]. With spaced training, the same amount of training as in massed training but interspersed with some periods of rest, both ARM and an LTM component are induced [4]. ARM and LTM are thought to exist in parallel, or the LTM is antagonistic to ARM several hours after training [4], [5]. Indeed, it has been proposed that *rsh* might be important for a pathway parallel to the cAMP / PKA pathway in memory formation [5]. In appetitive olfactory memory with a single training session, inducing maximal memory levels, and place memory with an extended intermittent training session, we may be inducing a memory that is strongly influenced by the so-called ARM component. This interpretation depends on the thus far perfect correlation between the effects of anesthesia on aversive olfactory memory and mutation of the *rsh* gene. The partial effects of *rsh^1^* on place memory and appetitive olfactory memory suggests a second component is also important, which could correspond to a *rutabaga* (*rut*) adenylyl cyclase function [23], [29]–[31]. Double mutant tests with these genes would address this possibility.

Why does aversive olfactory memory largely require the *rsh* component hours after conditioning but appetitive olfactory memory (more stable) and place memory (less stable) require *rsh* within minutes of training? Three possible explanations for the timing difference of the *rsh* memory component are explored. The first possible explanation is the complexity of the memories that are induced. The electric-shock reinforced olfactory memory is complex in the sense that it induces both an odorant approach and avoidance memory; the net odorant avoidance behavior of flies after this type of training is a combined effect of these memories. This complexity is revealed in altering the timing of shock / odor presentation and by genetic mutation [31]–[33]. There is no evidence for this sort of complexity in rewarded olfactory memory or place memory. A second possibility is the degree to which operant conditioning contributes to a memory. In aversive olfactory conditioning there should be very little if any operant learning as flies are presented with both odorants and electric shocks on a fixed timing schedule. In contrast, place memory should have a strong operant component since a fly learns about the space / temperature contingency by walking back and forth inside the heat-box chamber. Similarly, in rewarded olfactory learning flies presumably actively taste / ingest the sugar that is paired with the odorants during training (although the odorants are still surrounding the flies when they are not feeding too, so an operant component in this paradigm would require an emphasis on the pairing of feeding and perception of odorant). Third, an attention deficit with visual cues has been identified in *rsh^1^* mutant flies [34], which might influence the interpretation of the memory deficits of *rsh^1^* mutant flies described here. As much as one can extrapolate results from one test of attention onto different learning paradigms, it is possible that as the memories that are formed depend more on operant learning, an interaction of attention deficits and memory might give rise to a more severe memory deficit. Two sets of results argue against a major influence of *rsh*-dependent attention on memories tested here. Flies mutant for the *rsh* gene perform well in control experiments, which of course also require operant behaviors and, therefore, also likely require attention. Moreover, *rsh* mutant flies have largely normal learning in place memory and aversive olfactory memory (tests of appetitive olfactory memory at 3 min after training cannot directly address a learning deficit), suggesting that the attention in early phases of the experiments is sufficient for conditioning behavior. Altogether, it might be that the systems recruited in the more straight-forward memory forming conditions (i.e., one that does not elicit a mixture of approach and avoidance behavior to a stimulus that predicts the reinforcer) or with a significant operant component, establish the *rsh*-dependent phase earlier. In aversive olfactory memory, a delayed system recruits the *rsh*-dependent memory component.

The *rsh* memory component receives input from multiple sensory modalities and signal cascades. Aversive olfactory conditioning requires the dopaminergic system, and activation of these neurons paired with an odorant can be used to induce an aversive memory [23], [35]–[37]. In appetitive olfactory learning, the octopamine system is both necessary and sufficient for reinforcing this memory [23], [37]. Finally, in place learning, serotonin, but not dopamine or octopamine, are critical for memory formation [38], [39]. Each of these aminergic systems provide critical input to an associative process that in turn acts on a *rsh*-dependent consolidation [4], [13], [15], [16]. The different G-protein coupled receptor cascades that transduce these aminergic signals should eventually feed into the *rsh* pathway. Furthermore, since the neural structures for olfactory and place memory are different [7], [14], [30], [40], if the input to the *rsh* pathway is direct, *rsh* should be functioning in multiple parts of the fly brain. Alternatively, if *rsh* acts in an indirect fashion, there might be a single neural structure that requires *rsh* across different types of learning. Localized gene expression and behavioral rescue experiments will address these latter possibilities.

In conclusion, the *rsh* gene identifies a memory component that can be induced with different training regimens. *When* this component critically influences behavior depends on the learned task. It can influence memory performance from minutes to hours after training. Importantly, the *rsh* memory component does not scale with the stability of a memory trace. This suggests that what one ultimately measures as a change in behavior with training is the combined influence of multiple memory components, each of which has its own temporal property.

## Materials and Methods

### Flies and rearing conditions

Wild-type Canton S (CS), *radish^1^* (*rsh^1^*), and *rsh^1^*; hs- *rsh1*flies [12] were reared under standard conditions [31]. Flies with the *rsh^1^* allele had a *white*+ X-chromosome. Flies used for attempted appetitive olfactory memory rescue experiments were the male progeny of CS or *rsh^1^* female flies crossed with *w^1118^*; hs-*rsh1* male flies. Because heat-shocks of temperatures from 37 to 41°C for 15 to 40 min durations after starvation were deleterious to appetitive olfactory memory (not shown), no heat-shock was given prior to the behavioral experiments shown in Fig. 2C. Flies were between 2 and 7 days old for behavioral experiments.

### Behavioral experiments

Place learning used the heat-box [41]. Flies were trained as described in the results section or with intermittent training (three 6 min sessions with 3 min intervals) using 24/41°C temperatures [18], [19], [42]. Memory was tested for 3 min, either tested directly after conditioning or after an interval in which flies were held in fly food vials [20]. A 1 min reminder training was used to test memory after intermittent training. The measure of activity is the average probability of moving in the pre-test period [24].

Olfactory learning. Undiluted 4-methylcyclohexanol (MCH) and 3-octanol (OCT) were used as odorants with protocols previously described [16], [30]. To test for a potential *rsh* aversive olfactory memory deficit, memory was tested 3 min after training; for the 3 hr memory a 2 min cold-shock was presented 2 hrs after conditioning [4]. Flies were held in fly food vials in the longer retention intervals. Flies were trained by pairing either MCH or OCT with 12, 100 V electric shocks [30]. For appetitive memories, flies were tested at several time points after conditioning. Conditioning of flies was done after 16 to 20 hrs with access to only water by pairing MCH or OCT with 1 M sucrose dried on filter paper for 2 min (similar to) [16], the other odorant was paired with filter paper that was water treated and then dried. Flies were held in empty fly food vials in the retention intervals. Flies were given 1 min to choose between converging odorant streams in both the aversive and appetitive olfactory memory tests. Control experiments tested the ability of flies to sense and avoid the odorants used in the conditioning experiments against air, the testing period was again for 1 min [25], [30]. The ability of flies to sense sucrose was tested in vials with a stripe of sucrose (similar to) [23]. The proportion of flies on the stripe every 10 seconds was determined over 2 min, and the average over this period was used as a sucrose responsivity measure.

### Indices of behavior

For place learning, a Performance Index (PI) is used to calculate altered place preference [29]. Statistical tests use non-parametric Kruskal Wallis tests with Multiple Comparisons [28]. For olfactory learning, flies avoiding the odorant associated with shock (or approaching the odorant associated with sucrose) are used to generate an olfactory memory PI [3]. Olfactory memory PIs from flies of different genotypes are compared with an ANOVA and Newman-Keuls post-hoc tests [30].
